# A rare case of solitary plasmacytoma mimicking submucosal lesion of ascending colon: a case report and literature review

**DOI:** 10.3389/fonc.2024.1430745

**Published:** 2024-08-29

**Authors:** Alessandra Carella, Francesco Spannella, Sonia Morè, Riccardo Grifoni, Carlo Romano Settanni, Alessandra Mandolesi, Alessandra Filosa, Gaia Goteri, Gianfranco Boccoli, Giammarco Fava

**Affiliations:** ^1^ Unit of Gastroenterology and Digestive Endoscopy, Scientific Institute for Research, Hospitalization and Healthcare (IRCCS) Italian National Research Centres on Aging (INRCA), Ancona, Italy; ^2^ Internal Medicine and Geriatrics, Scientific Institute for Research, Hospitalization and Healthcare (IRCCS) Italian National Research Centres on Aging (INRCA), Ancona, Italy; ^3^ Department of Clinical and Molecular Sciences, University “Politecnica Delle Marche”, Ancona, Italy; ^4^ Hematology Clinic, Azienda Ospedaliero Universitaria delle Marche, Ancona, Italy; ^5^ Department of General Surgery, Scientific Institute for Research, Hospitalization and Healthcare (IRCCS) Italian National Research Centres on Aging (INRCA), Ancona, Italy; ^6^ Anatomic Pathology, Department of Biomedical Sciences and Public Health, University “Politecnica delle Marche”, Ancona, Italy

**Keywords:** plasmacytoma, gastrointestinal tumors, submucosal lesion, colonoscopy, digestive endoscopy

## Abstract

Solitary primary extraosseous plasmacytoma is a rare disease in the gastrointestinal tract, recently classified as an “exceptional” tumor of the colon site. The real incidence (one case/population/year) is unknown but reasonably less than 1/10,000,000 cases/year with very few descriptions in the literature. The rare cases described in the literature are often diagnosed after surgery for perforation and with predominant localization of the left colon. The main endoscopic presentation mimics colon carcinoma with ulcerated mass and obstructing lumen. In this paper, we report a rare case of isolated mass mimicking a submucosal lesion of the ascending colon diagnosed in an older female patient by colonoscopy. The patient was almost asymptomatic; she reported only a history of hematochezia without anemia. This mass was successfully treated by surgery and followed by hematological investigations, including bone marrow biopsy, specific laboratory tests, and CT/PET scan, which confirmed primary isolated plasmacytoma of the colon.

## 1 Introduction

Solitary primary extraosseous plasmacytoma is a rare disease in the gastrointestinal (GI) tract, recently classified as an “exceptional” tumor of the colon site (1, 2). The rare cases described in the literature are often diagnosed after surgery for perforation and with predominant localization of the left colon (2, 3). The main endoscopic presentation mimics colon carcinoma with ulcerated mass and obstructing lumen (4–6). In this paper, we describe a rare case of isolated submucosal mass of the ascending colon that resulted in solitary plasmacytoma in an older patient and which was successfully treated by surgery.

## 2 Case description

**Figure 1:**
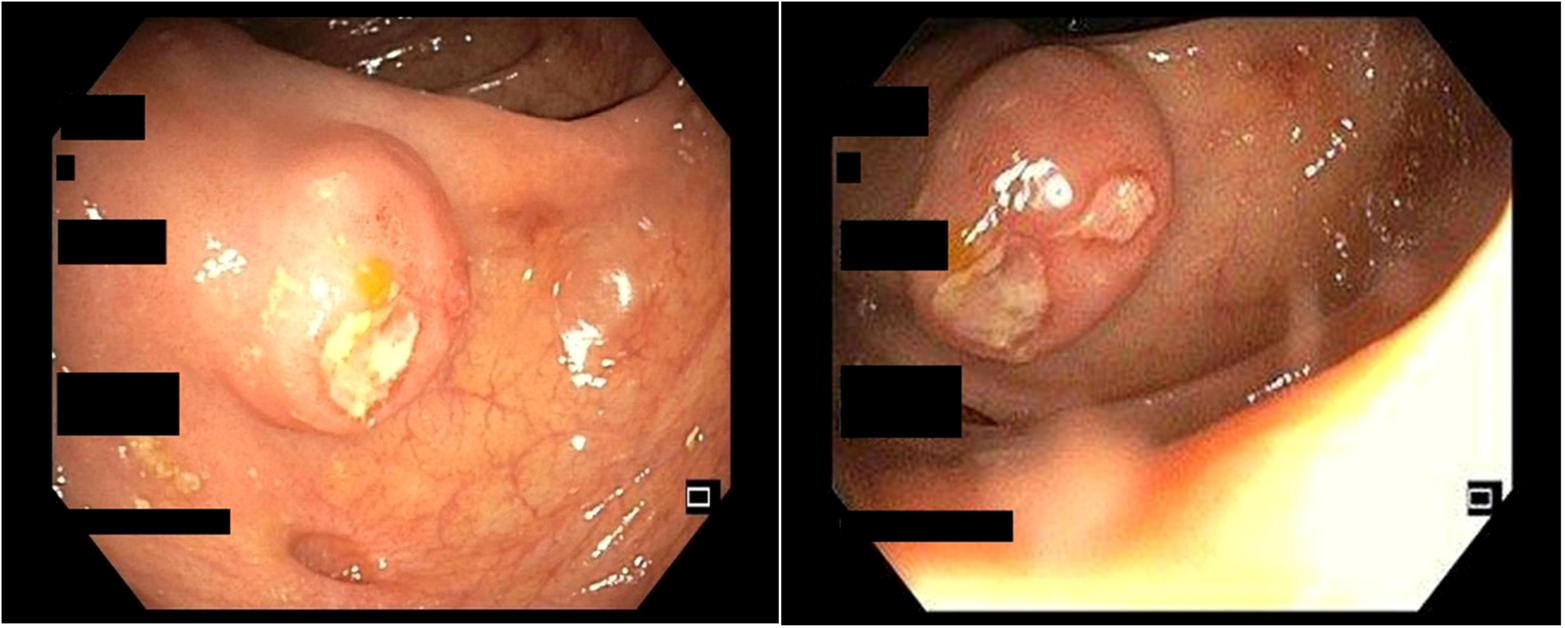
Colonoscopic view of a submucosal lesion in the ascending colon with two superficially ulcerated areas.

An 82-year-old Caucasian woman with a history of hematochezia by 1 month was admitted to our unit for a colonoscopy to be performed. She was asymptomatic, and the general physical examination was normal: her abdomen was soft, with no palpable masses, and not painful. The patient did not report weight loss. In her history, there was hypertension and dyslipidemia, a history of gastric ulcer, previous appendectomy, tonsillectomy, and knee prosthesis placement. She had no family history of colorectal cancer. We performed a full colonoscopy up to the cecum. In the ascending colon, near the ileocecal valve, we observed a nodular mass of about 4 cm mimicking a submucosal lesion covered by normal mucosa with two superficially ulcerated areas (**Figures 1A, B**). A colic gastrointestinal stromal tumor (GIST) was suspected, and multiple biopsies were performed mainly in the ulcerated parts. Histological examination showed a colonic mucosa infiltrated by cells with morphology and immunophenotype of the plasma cell (CD79a+, MUM1+, CD 138+, CD 20-, Bcl1-) with monotypic chain expression (IgA-k). Deposit of amyloid substance in the submucosa is observed.

**Figure 2:**
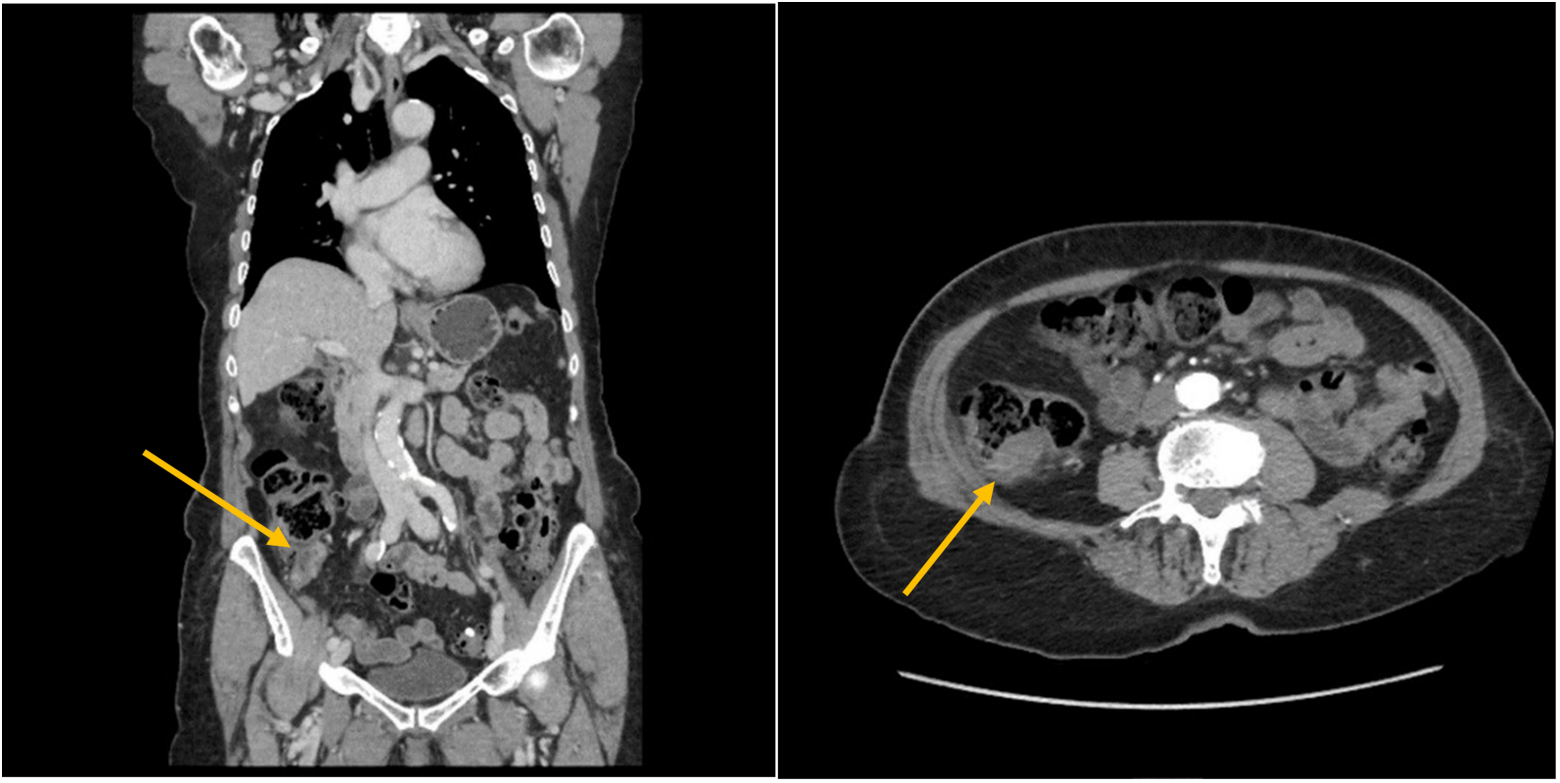
Abdominal CT scan view of a parietal tumor in the ascending colon without lymphadenomegaly or other pertinent abdominal alterations.

At subsequent investigations, the abdominal computed tomography (CT) scan showed the known parietal tumor in the ascending colon without lymphadenomegaly or other pertinent abdominal alterations (**Figures 2A, B**).

**Figure 3:**
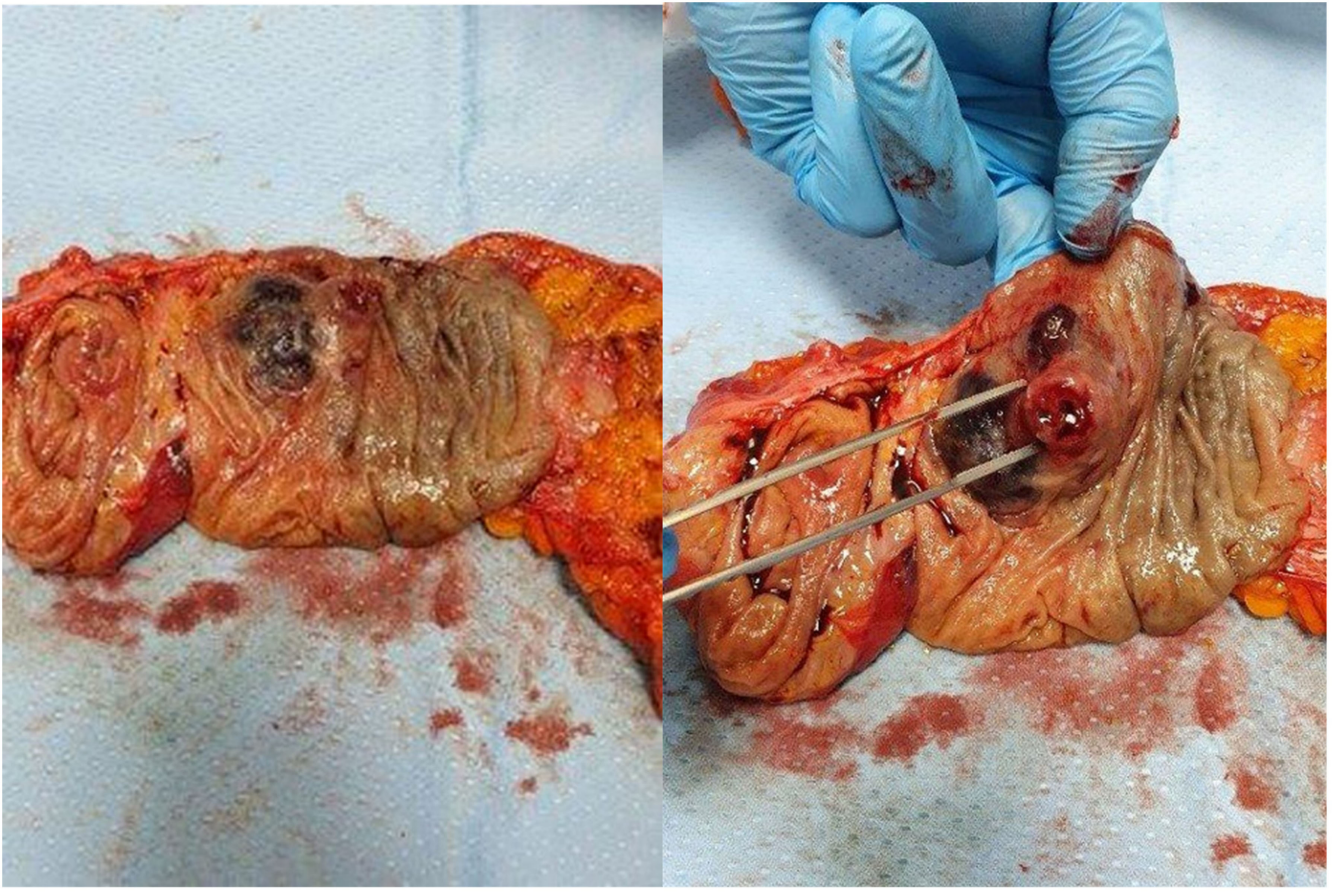
View of right hemicolectomy.

**Figure 4:**
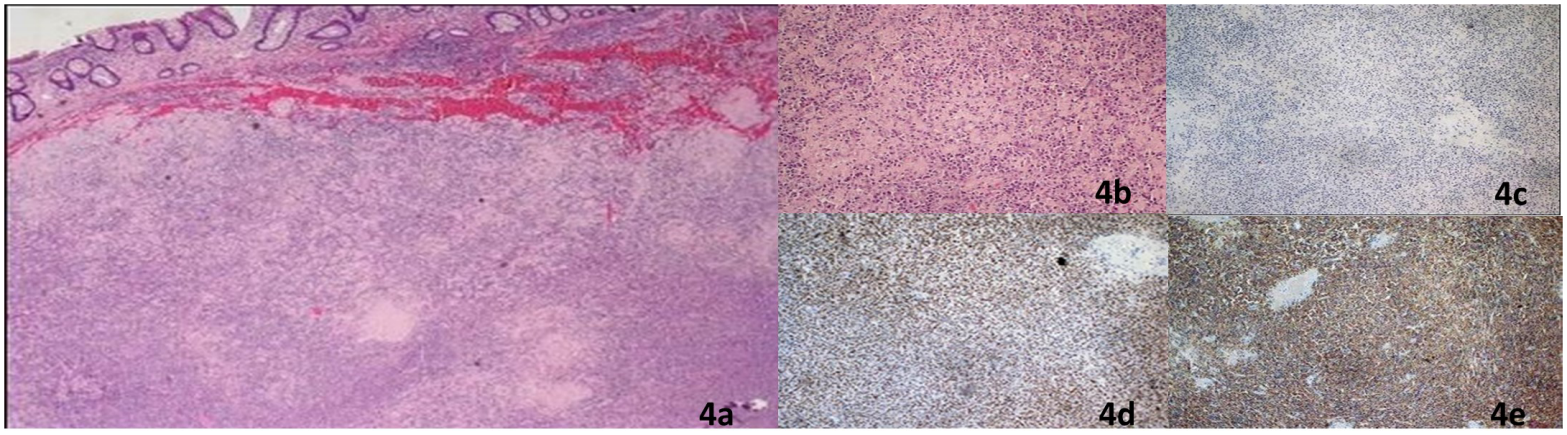
Histopathology of the surgical specimen: diffusely parietal infiltration by plasma cells with amyloid deposits **(A, B)** at immunohistochemistry, the plasma cells were CD20 negatives **(C)** and MUM1 positives **(D)** with a monotypic expression of IgA chains **(E)**.

The laboratory tests confirmed a good general condition without clear alterations of inflammatory biomarkers or anemia; the tumor markers and hepatic and renal function were normal. A right hemicolectomy was performed without adverse events (**Figures 3A, B**). The histopathological examination and immunohistochemistry study confirmed the previous result about plasma cell neoplasm (**Figure 4**). In particular, macroscopically, a 4.5-cm parietal nodule covered with ulcerated mucosa was identified in the ascendent colon. Microscopically, a neoplasm infiltrates the colon wall and perivisceral adipose tissue; the neoplasm is formed by cells with morphology and immunophenotype of a mature plasma cell (CD79a+, CD138+, MUM1+, CD20-, and Bcl1-) with monotypic expression of K light chains and IgA heavy chains. Deposit of amyloid substance is observed. The pericolic lymph nodes are reactive, without localization of the neoplasm (the details of the complete report are shown in the **Supplementary Materials**). The postoperative course was without complications, and the patient had good general condition at hospital discharge.

After surgery, hematologists performed a PET-CT scan to differentially diagnose solitary plasmacytoma vs. multiple myeloma, but it was negative for other systemic localizations of the disease and documented a complete remission of the unique GI localization. Laboratory serum urinary evaluation was negative, with serum monoclonal component not detected and negative serum immunofixation, urinary monoclonal component non-detected and negative urine immunofixation, and normal free light chain values with a normal ratio. A complete summary of the laboratory tests performed is shown in **Supplementary Table S1**. Bone marrow biopsy defined 40% cellularity without monoclonal plasma cells nor amyloid deposits (details of the complete report are shown in **Supplementary Materials**).

The last follow-up hematological visit was 21 months after surgery. Serum–urinary laboratory findings confirmed a hematological complete remission. The clinical conditions were good, without new hemorrhagic manifestations nor abdominal pain, defining an organ complete remission.

## 3 Discussion

Plasma cell neoplasms are a group of mature B-cell disorders. Like other hematological cancers, they are currently classified according to two different classifications, the 5th edition WHO and International Consensus Classification (ICC). According to the WHO classification, plasmacytoma is classified within the plasma cell neoplasms, together with plasma cell myeloma and plasma cell neoplasms with the associated paraneoplastic syndrome (7). According to ICC, plasma cell neoplasms are classified in non-IgM monoclonal gammopathy of undetermined significance (MGUS), multiple myeloma (plasma cell myeloma), solitary plasmacytoma of bone and extraosseous plasmacytoma, and the latter diagnoses in our case (8). Plasmacytoma, or its synonym extraosseous plasmacytoma in ICC classification, is a rare disease and accounts for <5% of plasma cell neoplasms. It is most often located in the head and neck region, mainly in the nasopharynx or upper respiratory tract in about 80% of cases; <10% occur in the GI tract (9). A recent review of literature on rare malignant tumors of the colon and rectum classified the plasmacytoma in the group of “exceptional” tumors of the colon and rectum (<1% of colorectal cancers) (2). The real incidence (one case/population/year) is unknown but reasonably less than 1/10,000,000 with very few descriptions in literature (2, 10). There are no markers able to differentiate plasma cells according to their origin. Therefore, plasmacytoma of the GI tract should be distinguished from systemic myeloma involving the GI tract through serological and imaging examinations associated with bone marrow biopsy. Recently, a rare case of plasma cell myeloma with ileal involvement with symptoms of GI obstruction and the presence of monoclonal IgA-k component has been described, as in our case (11). In our patient, PET-CT scan, laboratory tests, and bone marrow biopsy tested negative. Colon biopsies often reveal undifferentiated cells. Typical histological examination shows kappa light chain+, CD38+, CD3-, CD 20-, and CD 43- plasmacytoid cells (12). Kitamura et al. reported well-documented cases of plasmacytoma of the colon (in 2018) with a description of 30 from 1,928 cases, stratified on location, age, and clinical feature (3). Clinical presentation was variable and often reported with abdominal pain, intestinal bleeding, and diarrhea. Some cases were diagnosed by surgery after perforation (rectum and cecum) (3, 13). A case with multiple colonic strictures was also reported (12). Similar to our case, Kitamura et al. reported only two other cases of isolated mass in the ascending colon [Wing et al., 1975 (14); Doki et al., 2008 (15)] and in those cases diagnosed for pain and treated with hemicolectomy; in general, only five of well-documented cases of plasmacytoma were involved in over 80 patients (3). Overall, the localization of the left colon is described in the literature as predominant (2, 3). On endoscopic presentation, colonoscopy has not been described in these cases; it was not often completely performed for strictures. The mass presentation mainly mimicked colon carcinoma with ulcerated mass and obstructing lumen (2, 4–6). It may also mimic inflammatory bowel disease, and plasmacytoma may also coexist with Crohn’s disease (4, 12). In our case, the mass mimics a sub-mucosal lesion covered by normal mucosa with two superficially ulcerated areas. Presentation as a submucosal mass is even rarer (16, 17). Kodani et al. described a minute submucosal tumor (<10 mm) at the sigmoid colon that was successfully removed using endoscopic mucosal resection (16). Similarly, in another very recent case report, a polypoid submucosal lesion was found to be an isolated plasmacytoma in a 57-year-old asymptomatic man during screening colonoscopy (18). Previous cases of isolated colonic plasmacytoma and their main characteristics are summarized in **Supplementary Table S2**. The clinical and radiologic presentations are not specific. Because primary isolated plasmacytoma in the colon is very rare, the clinical course, treatment guidelines, and prognosis remain unclear. The available evidence supports a good prognosis with a 5-year survival of about 90% in localized disease and no lymph node involvement (19). Endoscopic treatments such as submucosal resection or polypectomy have proven to be sufficient in selected cases (16, 20). In our case, with localized disease and no lymph node involvement, surgical treatment appears curative.

## 4 Conclusion

Primary colonic plasmacytoma is a rare clinical entity. We described a rare case of plasmacytoma of the ascending colon mimicking the submucosal lesion. In our case, the prognosis was good and surgical treatment appears curative. Although these cases are rare, gastroenterologists, radiologists, hematologists, and pathologists should be aware of this entity.

## Data Availability

The raw data supporting the conclusions of this article will be made available by the authors, without undue reservation.
