# Supplementary material for: A rare case of solitary plasmacytoma mimicking submucosal lesion of ascending colon: a case report and literature review
Source: Front Oncol. 2024 Aug 29;14:1430745. doi: 10.3389/fonc.2024.1430745 (PMC11390380; doi:10.3389/fonc.2024.1430745)
Supplement: Supplementary file 1 [file DataSheet1.docx]

**The complete report of bone marrow biopsy**

Macroscopic description:

- Frustule of 1.3 cm and 0.07 g.

Microscopic description:

- Bone marrow biopsy with cellularity of approximately 40%, with good representation and maturation of the three haemophoresis series. There is no immunomorphological evidence of plasmacytoma localization in the clinical history. No evidence of amyloid substance deposits.

**The complete report of colon surgical specimens**

Macroscopic description:

- Right hemicolectomy including 6 cm ileum, 18 cm ascending colon and multiple fragments of omentum measuring 28 x 10 cm overall. At 5 cm from the ileocaecal valve, there is a nodule in the wall, which protrudes into the lumen, covered by mucosa, extending for 4.5 cm. When cut, the tumor has a greenish-white color.
- Loco-regional lymph nodes.

Microscopic description:

- Colonic wall and peri-colonic adipose tissue diffusely infiltrated by plasma cell neoplasm (CD79a+, CD 138+, MUM1+, CD20-, Bcl1-) with expression of IgA k chains. It is associated with amyloid deposition. The set of findings is consistent with the diagnosis of plasmacytoma.
- Reactive lymph nodes.

Supplemental Table S1. Complete laboratory tests.

| **Laboratory tests** | **Values** | **Normale ranges** |
| --- | --- | --- |
| **Urinary proteins** | 0.06 g/l |  |
| **24-hour urine proteins** | 0.12 g/24 h | 0.00-0.15 g/24 h |
| **Serum immunofixation** | No evident homogeneous monoclonal thickenings of immunoglobulins and/or light chains |  |
| **Free K light chains in serum** | 31,5 mg/dl | 3.3- 19.4 mg/dl |
| **Free L light chains in serum** | 26,5 mg/dl | 5.7-26.3 mg/dl |
| **Serum-free k/l ratio** | 1.18 | 0.26-1.65 |
| **IgG** | 1710 mg/dl | Up to 1 year 230-1000 mg/dl |
| **IgA** | 194 mg/dl | Up to 1 year 5-140 mg/dl |
| **IgM** | 57 mg/dl | Up to 1 year 8-70 mg/dl |
| **Total bilirubin** | 0.80 mg/dl | 0.20-1.20 mg/dl |
| **Direct bilirubin** | 0.20 mg/dl | 0.00-0.40 mg/dl |
| **Indirect bilirubin** | 0.60 mg/dl | 0.00-0.80 mg/dl |
| **Aspartate aminotransferase (AST)** | 20 U/l | 0-40 U/l |
| **Alanine Aminotransferase (ALT)** | 28 U/l | 0-40 U/l |
| **Lactate dehydrogenase (LDH)** | 196 U/l | Up to 16 years 325 U/l |
| **Alkaline phosphatase (ALP)** | 61 U/l | Up to 1 year 0-550 U/l |
| **GT gamma (GGT)** | 12 U/l | 4-45 U/l |
| **Serum calcium** | 8.9 mg/dl | 8.5-10.5 mg/dl |
| **Serum sodium** | 138 mEq/l | 136-148 mEq/l |
| **Serum potassium** | 3.9 mEq/l | 3.6-4.9 mEq/l |
| **Serum protein electrophoresis** | See graph below |  |
| **Albumin %** | 53% | 55.8-66.1% |
| **Alpha 1%** | 4.1 % | 2.9-4.9% |
| **Alpha 2%** | 9.3% | 7.1-11.8% |
| **Beta 1%** | 6.5% | 4.7-7.2% |
| **Beta2%** | 5.0% | 3.2-6.5% |
| **Gamma %** | 22.1% | 11.1-18.8% |
| **Albumin #** | 4.08 g/dl | 4.02-4.76 g/dl |
| **Alpha1#** | 0.32 g/dl | 0.21-0.35 g/dl |
| **Alpha2#** | 0.72 g/dl | 0.51-0.85 g/dl |
| **Beta1 #** | 0.50 g/dl | 0.34-0.52 g/dl |
| **Beta2#** | 0.39 g/dl | 0.23-0.47 g/dl |
| **Gamma#** | 1.70 g/dl | 0.80-1.35 g/dl |
| **A/G ratio** | 1.13 | 1.10-1.90 |
| **Comment** | The electrophoretic trace does not present visible alterations referable to monoclonal components |  |
| **Kappa urinary light chains** | 0.85 mg/dl | 0.00-0.85 mg/dl |
| **Lambda urinary light chains** | 0.47 mg/dl | 0.00-0.47 mg/dl |
| **Bence Jones proteins** | negative |  |
| **Erythrocytes red blood cells** | 4.40 x 10^3/microL | 4.00-5.00 x 10^3/microL |
| **Leukocytes (WBC)** | 8.48 x 10^3/microL | 4.00-10.00 x 10^3/microL |
| **Hemoglobin (Hb)** | 13.3 g/dl | 11.5-16.0 g/dl |
| **Platelets (PLT)** | 259 x 10^3/microL | 150-400 |
| **Hematocrit (HCT)** | 42.0 % | 36.0-44.0% |
| **Neutrophils** | 4.05 x 10^3/microL | 1.90-7.70 x 10^3/microL |
| **Lymphocytes** | 2.60 x 10^3/microL | 1.00-4.00 x 10^3/microL |
| **Monocytes** | 0.67 x 10^3/microL | 0.10-1.00 x 10^3/microL |
| **Eosinophils** | 1.09 x 10^3/microL | 0.01-0.50 x 10^3/microL |
| **Basophils** | 0.06 x 10^3/microL | 0.00-0.10 x 10^3/microL |
| **Erythrocyte sedimentation rate** | 41 mm/h | 0-38 mm/h |
| **INR** | 0.93 | 0.80-1.25 |
| **APTT** | 26 sec |  |
| **Urea** | 46 mg/l | 20-45 mg/l |
| **Serum uric acid** | 2.3 mg/dl | 2.5-7.0 mg/dl |
| **Carcinoembryonic antigen (CEA)** | 5.4 mcg/l | 0-5 mcg/l |
| **CA 19.9** | 15 U/l | 0-34 U/l |

**
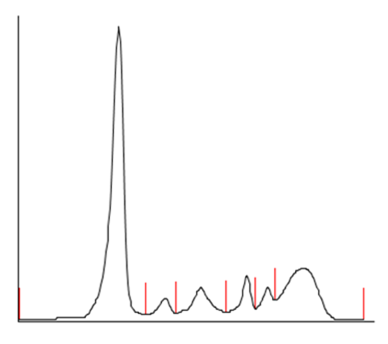
Graph of serum protein electrophoresis.**

Supplemental Table S2. Previous case reports of isolated colonic plasmacytoma and their main characteristics.

| **Author** | **Journal** | **Year of publication** | **Sex** | **Age (years)** | **Location** | **Clinical features** | **Therapy** |
| --- | --- | --- | --- | --- | --- | --- | --- |
| Hampton et al. | Ann Surg  . | 1957 | Female | 43 | Rectum | Rectal pain and bleeding | Surgery |
| Miller W.A. | J Can Assoc Radiol  . | 1970 | M | 35 | Cecum | Anemia | Surgery |
| Wing et al. | JAMA | 1975 | Female | 82 | Ascending colon | Pain | Surgery |
| Shaw et al. | Cancer | 1976 | Female | 47 | Cecum | Diarrhea | Surgery |
| Allison et al. | S Afr Med J | 1977 | Male | 61 | Sigmoid colon | None | Surgery |
| Adekunle O.O. | J R Coll Surg Edinb | 1978 | Male | 35 | Cecum | Pain | Surgery |
| Sidani et al. | Dis Colon Rectum  . | 1983 | Male | 52 | Sigmoid colon | Pain, rectal bleeding | Surgery |
| Ligato et al. | Arch Pathol Lab Med | 1996 | Male | 45 | Right colon | Anemia | Surgery |
| Holland et al. | J R Coll Surg Edinb | 1997 | Male | 62 | Sigmoid colon | Pain | Surgery |
| Lattuneddu et al. | Int J Colorectal Dis | 2004 | Male | 86 | Sigmoid colon | Pain, rectal bleeding | Surgery |
| Gupta et al. | World J Surg Oncol. | 2007 | Male | 42 | Diffuse colon | Diarrhea | Surgery |
| Jones et al. | Am Surg. | 2008 | Male | 65 | Sigmoid colon | Pain | Surgery |
| Doki et al. | Int J Colorectal Dis | 2008 | Male | 64 | Ascending colon | Pain | Surgery |
| Collado Pacheco et al. | Endoscopy | 2009 | Male | 74 | Right colon | Pain, rectal bleeding | ? |
| Kodani et al. | Endoscopy | 2011 | Male | 42 | Sigmoid colon | Fecal occult blood | Endoscopic resection |
| Nakagawa et al. | Endoscopy | 2011 | Female | 84 | Cecum and rectum | Medical examination | Endoscopic resection |
| Lee et al. | Gastroenterology Res. | 2013 | Male | 45 | Transverse colon | Pain | Surgery |
| Zihni et al. | Ulus Cerrahi Derg. | 2013 | Male | 54 | Descending colon | Pain and weakness | Surgery |
| Han et al. | Korean J Gastroenterol | 2014 | Male | 49 | Transverse colon | Pain | Surgery |
| Parnel et al. | BMJ Case Rep. | 2015 | Female | 72 | Right colon | Fatigue, dark stool | Surgery |
| Zhu et al. | Clin Gastroenterol Hepatol | 2017 | Female | 67 | Ascending colon | Pain | Chemotherapy/death |
| Kitamura et al | Surg Case Rep | 2018 | Female | 77 | Sigmoid colon | Pain | Surgery |
| Bhangoo et al. | Dig Liver DIs | 2021 | Male | 82 | Rectus Sigmoid | Rectal bleeding | RT/Surgery |
| Nassif et al. | ACG Case Rep J | 2023 | Male | 57 | Sigmoid colon | Asymptomatic | Endoscopic resection |
|  |  |  |  |  |  |  |  |
|  |  |  |  |  |  |  |  |
